# Overall survival by baseline and on-treatment systemic immune-inflammation index in patients with advanced cancer receiving immune checkpoint inhibitors: a large single-centre cohort study

**DOI:** 10.1093/immadv/ltaf031

**Published:** 2026-02-27

**Authors:** Oliver John Kennedy, Rebecca Lee, Fiona Blackhall, Ananya Choudhury, Robert Metcalf, Tom Waddell, Paul Lorigan

**Affiliations:** Division of Cancer Sciences, University of Manchester, Oxford Rd, Manchester, M13 9PL, United Kingdom; Christie NHS Foundation Trust, Wilmslow Rd, Manchester, M20 4BX, United Kingdom; Division of Cancer Sciences, University of Manchester, Oxford Rd, Manchester, M13 9PL, United Kingdom; Christie NHS Foundation Trust, Wilmslow Rd, Manchester, M20 4BX, United Kingdom; Division of Cancer Sciences, University of Manchester, Oxford Rd, Manchester, M13 9PL, United Kingdom; Christie NHS Foundation Trust, Wilmslow Rd, Manchester, M20 4BX, United Kingdom; Division of Cancer Sciences, University of Manchester, Oxford Rd, Manchester, M13 9PL, United Kingdom; Christie NHS Foundation Trust, Wilmslow Rd, Manchester, M20 4BX, United Kingdom; Christie NHS Foundation Trust, Wilmslow Rd, Manchester, M20 4BX, United Kingdom; Division of Immunology, Immunity to Infection and Respiratory Medicine, University of Manchester, Manchester, United Kingdom; Christie NHS Foundation Trust, Wilmslow Rd, Manchester, M20 4BX, United Kingdom; Division of Cancer Sciences, University of Manchester, Oxford Rd, Manchester, M13 9PL, United Kingdom; Christie NHS Foundation Trust, Wilmslow Rd, Manchester, M20 4BX, United Kingdom

**Keywords:** immunotherapy, immune checkpoint inhibitors, Systemic Immune-Inflammation Index, overall survival, lung cancer, melanoma

## Abstract

The Systemic Immune-Inflammation Index (SIII; neutrophils/lymphocytes × platelets) is a low-cost biomarker proposed to predict outcomes with immune checkpoint inhibitors (ICIs). This study evaluated associations of baseline and early on-treatment changes in SIII with overall survival (OS) for common ICI regimens. Patients with advanced cancer treated with ICIs at a UK centre were categorized by baseline SIII (above vs. below the median) and by changes at 3–6 weeks (increase/decrease). OS was analysed using Kaplan–Meier estimates. Adjusted hazard ratios (aHRs) with 95% confidence intervals (CIs) were calculated using multivariable Cox regression. Among 2578 patients included, 1514 deaths occurred over a median follow-up of 2.6 years. Common regimens included pembrolizumab or atezolizumab with (15.9%) or without chemotherapy (13.9%) for NSCLC, and nivolumab plus ipilimumab for melanoma (12.6%). Lower baseline SIII was associated with improved OS (28.1 vs. 11.1 months; aHR 0.56, 0.50–0.62), with a weaker association observed in those receiving ICI-targeted therapy combinations. An on-treatment increase in SIII was linked to reduced OS (16.8 vs. 21.5 months; aHR 1.33, 1.18–1.49). Patients with low baseline SIII and an on-treatment decline had the longest OS (33.2 months), whereas those with high baseline SIII and an on-treatment increase had the shortest (8.2 months; aHR 2.88, 2.41–3.44; interaction between baseline and on-treatment SIII *P* < 0.001). SIII is a low-cost, readily available biomarker. Both baseline SIII levels and on-treatment changes in SIII are significantly associated with OS. SIII may help identify patients who could benefit from closer monitoring or treatment adjustments.

## Introduction

Immune checkpoint inhibitors (ICIs) are used in the neoadjuvant, adjuvant, and advanced settings across a wide range of cancers and have revolutionized clinical outcomes [1–6]. They function by stimulating the immune system to target and destroy cancer cells [7]. However, despite their success, ICIs are not effective in a subset of patients [8]. Better identification of these patients is needed to guide alternative treatment strategies or enable closer monitoring for treatment failure. The development of effective biomarkers that would enable such patients to be identified remains an area of ongoing interest [9].

The Systemic Immune-Inflammation Index (SIII) has been proposed as a potential biomarker for ICI therapy [10]. SIII is readily calculated from routine blood tests (neutrophils/lymphocytes × platelets). A higher SIII may indicate an inflammatory state, which could promote tumour progression, suppress anti-tumour immunity, and reduce the effectiveness of ICIs. While previous studies have suggested associations between SIII and survival, its relationship with outcomes across commonly used ICI regimens in routine care remains unclear.

In this study, we evaluated the association of baseline pre-treatment SIII levels and early on-treatment changes in SIII with OS in ICI-based regimens commonly used in clinical practice at a large UK cancer centre.

## Materials and methods

This retrospective cohort study included patients treated for advanced cancer at The Christie NHS Foundation Trust, Manchester, UK, between 2018 and 2023. The Christie is a large cancer centre serving a population of 3.2 million people, primarily in the North West of England. It provides treatment to over 60 000 patients each year for a broad range of solid organ and haematological malignancies [11]. In this study, all patients receiving commonly used standard-of-care ICI regimens were included. Data on age, sex, performance status, cancer type, treatment regimen, and blood tests were extracted from electronic patient records.

Regimens included, for non-small cell lung cancer (NSCLC), first-line treatment with pembrolizumab or atezolizumab combined with chemotherapy, as well as monotherapy with either agent in first- or second-line settings. In melanoma, patients received either nivolumab and ipilimumab or monotherapy with nivolumab or pembrolizumab. For head and neck cancers, nivolumab or pembrolizumab was used in first- or second-line settings. Small-cell lung cancer was treated with first-line atezolizumab in combination with chemotherapy. In renal cancer, regimens included first-line combinations of avelumab or pembrolizumab with a tyrosine kinase inhibitor (TKI), first-line nivolumab and ipilimumab, or second-line nivolumab monotherapy. Urothelial carcinoma patients received second-line atezolizumab or pembrolizumab.

As part of their standard-of-care treatment, patients underwent routine blood testing within 48 hours prior to treatment initiation, followed by additional tests within 48 hours of each subsequent treatment cycle. Cycle lengths varied by regimen, ranging from a minimum of three weeks to a maximum of six weeks. Additional blood tests were performed when clinically indicated, as directed by the treating clinician. For the blood test immediately before cycle 1 and for the first blood test 3–6 weeks after cycle 1, SIII was calculated as (neutrophil count/lymphocyte count) × platelet count.

Patients were categorized using three distinct SIII-based classifications. The first classification was similar to previous comparable studies and based on pre-treatment SIII, dichotomized as above or below the median across all patients (or by subgroup) at treatment initiation [12]. The second classification was also similar to previous studies [13] and was according to on-treatment SIII changes, assessed 3–6 weeks after treatment initiation, and categorized as an increase or decrease relative to each patient’s individual baseline value (i.e. on-treatment increase or decrease). The third classification combined both baseline and on-treatment SIII values, creating four distinct groups: baseline SIII below the median with on-treatment fall, baseline SIII below the median with on-treatment rise, baseline SIII above the median with on-treatment fall, and baseline SIII above the median with on-treatment rise.

Median and 1-year OS according to baseline and on-treatment SIII were estimated using the Kaplan–Meier method, with survival distributions compared using the log-rank test. Multivariable Cox regression was used to estimate adjusted hazard ratios (aHRs) for associations between baseline SIII, early on-treatment SIII changes, and OS. The Cox model included terms to adjust for age, sex, performance status, and the combination of cancer type and treatment regimen. A separate model included an interaction term to assess whether the effect of on-treatment SIII changes on OS differed by baseline SIII category. Because ICIs are commonly combined with chemotherapy in NSCLC and with tyrosine kinase inhibitors in renal cancer, an additional interaction test was performed to assess whether treatment type modified the baseline SIII–OS association in these tumour groups.

Subgroup analyses were performed according to ICI regimen. Follow-up was estimated using the reverse Kaplan–Meier method. Statistical significance was defined as *P* < 0.05, and estimates were calculated with 95% confidence intervals. All analyses were conducted using R version 4.4.0. As no patient identifiable data was included in the analysis, individual patient consent was not required for this study. The study was approved by the Clinical Outcomes & Data Unit at The Christie NHS Foundation Trust (application number 4329).

## Results

A total of 2578 patients were included, with 1514 OS events occurring over a median follow-up of 2.6 years. Baseline patient characteristics are summarized in Table 1. The most common treatment regimens were pembrolizumab or atezolizumab with chemotherapy (n = 410, 15.9%) or without chemotherapy (n = 358, 13.9%) as first-line treatment for non-small cell lung cancer (NSCLC), followed by nivolumab plus ipilimumab for advanced melanoma (n = 324, 12.6%). The least common regimen was atezolizumab or pembrolizumab as second-line treatment for advanced bladder cancer (n = 125, 4.8%). For the entire cohort, 1-year OS was 59.3% (57.3–61.3%), and median OS was 17.1 months (16.0–18.7).

**Table 1.** Patient characteristics by baseline SIII and on-treatment SIII.

		Baseline SIII		On-treatment SIII	
	Total	Above median	Below median	Rising	Falling
**All**	2578	1289	1289	1124	1177
**Sex**					
Female	1004	540 (41.9)	464 (36.0)	418 (37.2)	466 (39.6)
Male	1574	749 (58.1)	825 (64.0)	706 (62.8)	711 (60.4)
**Age**					
<50	195	91 (7.1)	104 (8.1)	81 (7.2)	90 (7.6)
50–64	853	456 (35.4)	397 (30.8)	367 (32.7)	397 (33.7)
>65	1530	742 (57.6)	788 (61.1)	676 (60.1)	690 (58.6)
**ECOG PS**					
0	1435	641 (49.7)	794 (61.6)	648 (57.7)	667 (56.7)
1	993	552 (42.8)	441 (34.2)	409 (36.4)	461 (39.2)
2+	114	76 (5.9)	38 (2.9)	48 (4.3)	36 (3.1)
Unknown	36	20 (1.6)	16 (1.2)	19 (1.7)	13 (1.1)
**Blood counts**					
Median platelets (IQR)	298 (156)	367 (173)	255 (95)	279 (130)	319 (159)
Median neutrophils (IQR)	5.8 (3.5)	7.6 (3.8)	4.6 (2.0)	5.1 (2.8)	6.2 (4.0)
Median lymphocytes (IQR)	1.3 (0.9)	1.0 (0.7)	1.6 (0.8)	1.4 (0.9)	1.3 (0.8)
Median SIII (IQR)	1317 (1829)	2573 (2284)	743 (462)	1015 (1265)	1589 (2113)
**Regimen**					
**NSCLC**					
Pembrolizumab/atezolizumab with ChT (first line)	410 (15.9)	280 (21.7)	130 (10.1)	136 (12.1)	227 (19.3)
Atezolizumab/pembrolizumab (first line)	358 (13.9)	232 (18.0)	126 (9.8)	122 (10.9)	186 (15.8)
Atezolizumab/pembrolizumab (2nd line)	204 (7.9)	103 (8.0)	101 (7.8)	102 (9.1)	77 (6.5)
**Melanoma**					
Nivolumab and Ipilimumab (first or second line)	324 (12.6)	109 (8.5)	215 (16.7)	157 (14.0)	139 (11.8)
Nivolumab/pembrolizumab (1st or second line)	273 (10.6)	74 (5.7)	199 (15.4)	133 (11.8)	103 (8.8)
**Head and neck**					
Nivolumab/pembrolizumab (first or second line)	251 (9.7)	170 (13.2)	81 (6.3)	130 (11.6)	92 (7.8)
**SCLC**					
Atezolizumab with ChT (first line)	164 (6.4)	83 (6.4)	81 (6.3)	51 (4.5)	102 (8.7)
**Renal**					
Avelumab/pembrolizumab with TKI (first line)	168 (6.5)	62 (4.8)	106 (8.2)	57 (5.1)	101 (8.6)
Nivolumab (second line)	158 (6.1)	60 (4.7)	98 (7.6)	109 (9.7)	39 (3.3)
Nivolumab and ipilimumab (first line)	143 (5.5)	55 (4.3)	88 (6.8)	71 (6.3)	64 (5.4)
**Urothelial**					
Atezolizumab/pembrolizumab (second line)	125 (4.8)	61 (4.7)	64 (5.0)	56 (5.0)	47 (4.0)

ChT, chemotherapy; ECOG PS, Eastern Cooperative Oncology Group performance status; IQR, interquartile range; NSCLC, non-small cell lung cancer; SCLC, small cell lung cancer; SIII, systemic immune-inflammation index; TKI, tyrosine kinase inhibitor.

The median SIII among all patients was 1316 (Supplementary Table S1). In the below-median baseline SIII group (compared to the above-median group), there were higher proportions of male patients (64.0% vs. 58.1%) and those with an Eastern Cooperative Oncology Group performance status of 0 (61.6% vs. 49.7%). The below median baseline SIII group had more patients with favourable diagnoses, including melanoma (32.1% vs. 14.2%) and renal cancer (22.7% vs. 13.7%), and fewer patients with less favourable diagnoses, such as NSCLC (27.7% vs. 47.8%) and head and neck cancer (6.3% vs. 13.2%). The median age was similar between the two groups (67.3 vs. 68.9 years).

**Figure 1.**
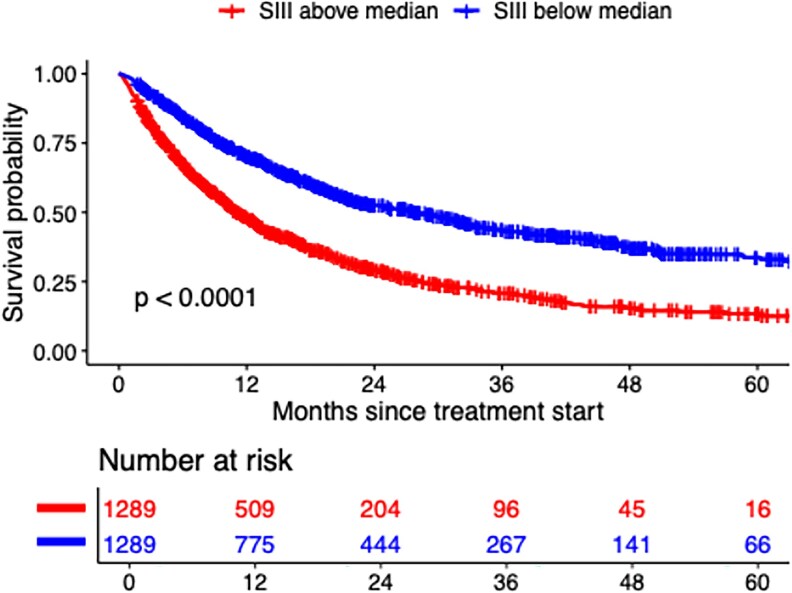
OS according to baseline systemic immune-inflammation index among patients receiving immune checkpoint inhibitors.

**Figure 2.**
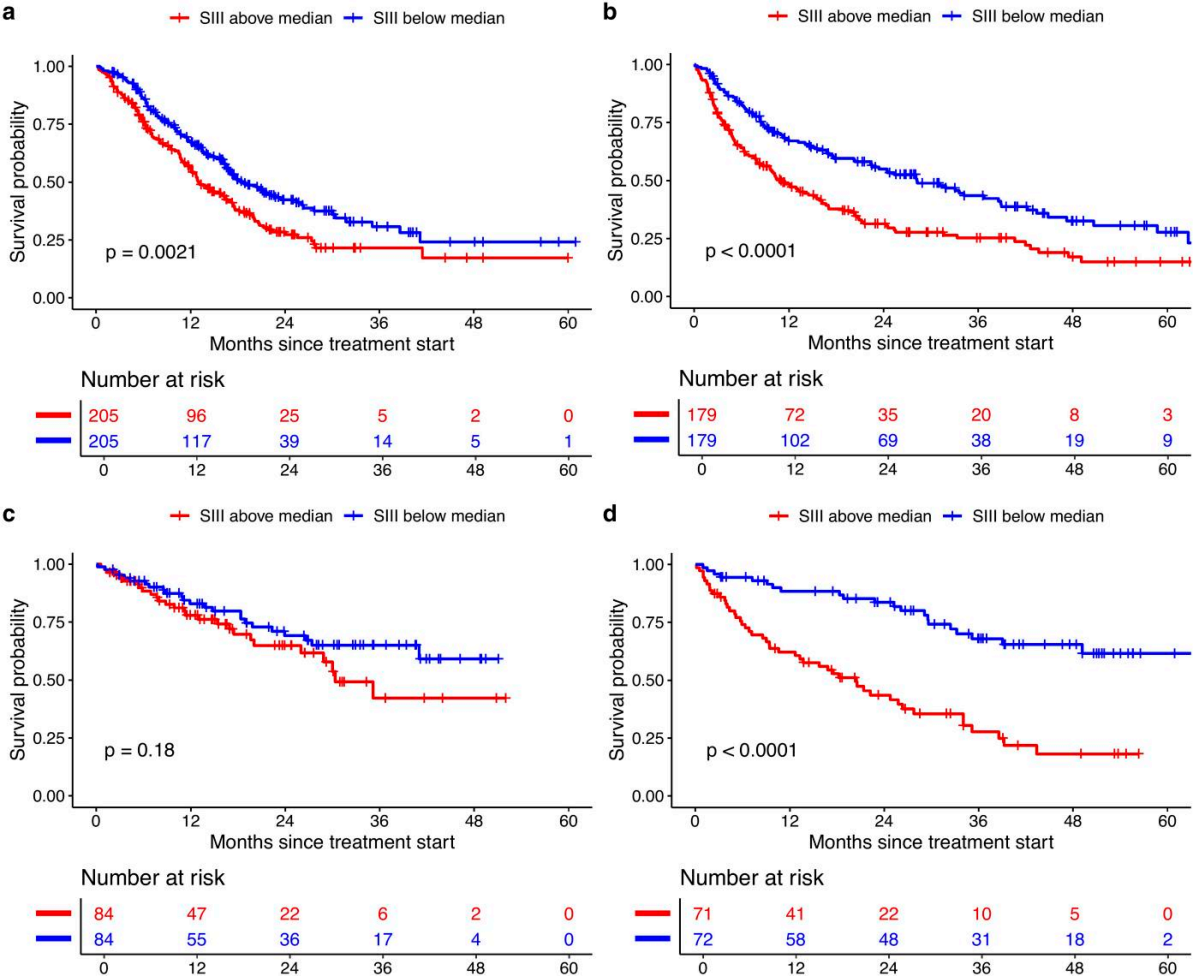
Overall survival by baseline systemic immune-inflammation index (SIII; above vs. below the median) in patients receiving first-line therapy: (a) NSCLC, pembrolizumab/atezolizumab + chemotherapy; (b) NSCLC, pembrolizumab/atezolizumab monotherapy; (c) renal cell carcinoma, avelumab/pembrolizumab + TKI; (d) renal cell carcinoma, nivolumab + ipilimumab.

As shown in Table 2 and Fig. 1, patients with below-median baseline SIII had longer OS than those with above-median SIII (1-year OS 70.1% vs. 48.3%; median OS 28.1 vs. 11.1 months; aHR 0.56, 0.50–0.62). This association was observed across most cancer types and treatment regimens (Fig. 2 and Supplementary Figures S1–S7). In renal cancer, it was significant for first-line nivolumab plus ipilimumab (aHR 0.24, 0.14–0.40) and second-line nivolumab (aHR 0.42, 0.29–0.63) but not for ICIs with a tyrosine kinase inhibitor (aHR 0.74, 0.42–1.29). In NSCLC, the association was present across all regimens, with a greater OS difference for ICI monotherapy (17.2 months) than for ICIs plus chemotherapy (5.6 months). Among patients with NSCLC and below-median SIII, OS was longer with ICI monotherapy than with ICI plus chemotherapy (28.2 vs. 18.7 months), while the opposite trend was seen for those with above-median SIII (13.1 vs. 11.0 months). Interaction tests were performed to evaluate whether the association between baseline SIII and OS was modified by the addition of chemotherapy in NSCLC and tyrosine kinase inhibitors in renal cancer; no significant interactions were observed, though these analyses may have been limited by sample size and patient heterogeneity.

**Table 2.** Overall survival by baseline SIII.

	Baseline SIII						
	Below median			Above median			aHR below vs. above median (95% CI)
Regimen	N/events	1-year OS (%)	Median OS (months)	N/events	1-year OS (%)	Median OS (months)	
**All**	1289/643	70.1 (67.6–72.8)	28.1 (23.5–31.9)	1289/871	48.3 (45.5–51.2)	11.1 (10.2–12.5)	0.56 (0.50–0.62)
**NSCLC**							
Pembrolizumab/atezolizumab with ChT (first line)	205/106	67.1 (60.7–74.2)	18.7 (16.5–26.2)	205/129	56.0 (49.3–63.6)	13.1 (11.7–16.8)	0.67 (0.52–0.87)
Atezolizumab/pembrolizumab (first line)	179/96	67.1 (60.3–74.6)	28.2 (22.5–38.9)	179/123	48.5 (41.5–56.7)	11.0 (8.3–15.9)	0.57 (0.44–0.75)
Atezolizumab/pembrolizumab (second line)	102/80	58.7 (49.7–69.2)	14.3 (11.5–20.4)	102/94	33.2 (25.2–43.9)	7.8 (6.2–9.9)	0.58 (0.42–0.79)
**Melanoma**							
Nivolumab and Ipilimumab (first or second line)	162/51	80.4 (74.3–87.0)	NA (NA-NA)	162/77	57.9 (50.4–66.6)	22.4 (12.5-NA)	0.54 (0.37–0.78)
Nivolumab/pembrolizumab (first or second line)	137/57	78.6 (71.8–86.0)	46.9 (27.1 to NA)	136/85	61.0 (53.1–70.0)	18.4 (14.2–29.9)	0.49 (0.34–0.69)
**Head and neck**							
Nivolumab/Pembrolizumab (first or second line)	126/84	50.2 (41.8–60.3)	12.5 (9.8–14.9)	125/98	31.3 (23.8–41.1)	6.5 (4.6–9.1)	0.62 (0.46–0.83)
**SCLC**							
Atezolizumab with ChT (first line)	82/57	53.0 (42.8–65.6)	13.5 (9.9–15.5)	82/58	36.6 (26.5–50.5)	8.9 (6.9–12.2)	0.58 (0.39–0.85)
**Renal**							
Avelumab/pembrolizumab with TKI (first line)	84/24	82.9 (74.8–91.9)	NA (41.0-NA)	84/27	77.9 (68.9–88.2)	30.4 (26.0-NA)	0.74 (0.42–1.29)
Nivolumab (second line)	79/43	73.5 (64.2–84.2)	25.5 (17.9–44.6)	79/67	44.2 (34.3–56.9)	10.2 (7.1–15.6)	0.42 (0.29–0.63)
Nivolumab and ipilimumab (first line)	72/21	88.4 (81.1–96.3)	NA (49.2-NA)	71/47	62.1 (51.6–74.8)	20.5 (13.3–34.0)	0.24 (0.14–0.40)
**Urothelial**							
Atezolizumab/pembrolizumab (second line)	63/41	48.1 (36.6–63.2)	8.0 (7.4–30.6)	62/49	26.8 (17.2–41.7)	6.0 (4.0–9.7)	0.60 (0.38–0.95)

aHR, adjusted hazard ratio; ChT, chemotherapy; CI, confidence interval; NSCLC, non-small cell lung cancer; OS, overall survival; SCLC, small cell lung cancer; SIII, systemic immune-inflammation index; TKI, tyrosine kinase inhibitor.

Baseline SIII was strongly associated with OS both for ICI monotherapy and combination therapy in melanoma (aHR 0.54, 0.37–0.78 for nivolumab plus ipilimumab; aHR 0.49, 0.34–0.69 for nivolumab or pembrolizumab monotherapy). Significant associations were also observed for ICIs combined with chemotherapy in small cell lung cancer (aHR 0.58, 0.39–0.85), as well as for first- and second-line ICIs in head and neck cancer (aHR 0.62, 0.46–0.83) and second-line ICIs in bladder cancer (aHR 0.60, 0.38–0.95).

Patients with an on-treatment fall in SIII (compared to a rise) had a longer median OS (21.5 months, 19.9–25.2 vs. 15.7 months, 13.8–17.6) and higher 1-year OS (68.9%, 66.1–71.7 vs. 56.1%, 53.1–59.2), with an aHR of 1.36, 1.22–1.53 (Table 3). The effects were most pronounced in bladder cancer (median OS 26.8 months, 12.8–38.6 vs. 6.1 months, 4.2–8.2; 1-year OS 64.9%, 51.5–81.8 vs. 27.7%, 17.8–43.2; aHR 2.88, 1.71–4.86) and head and neck cancer (median OS 13.9 months, 11.7–18.4 vs. 8.1 months, 6.4–10.5; 1-year OS 59.4%, 49.8–70.8 vs. 32.8%, 25.2–42.8; aHR 1.84, 1.32–2.58). In first-line NSCLC, the association was significant for ICI without chemotherapy (aHR 1.67, 1.24–2.25) but not for ICI with chemotherapy (aHR 1.14, 0.86–1.53). In melanoma, the association did not reach significance for either nivolumab plus ipilimumab (aHR 1.19, 0.81–1.74) or nivolumab/pembrolizumab monotherapy (aHR 1.17, 0.81–1.71), and no significant associations were observed in renal cancer.

**Table 3.** Overall survival by SIII at 3–6 weeks after treatment initiation.

	On-treatment SIII						
	Rising			Falling			aHR rising vs. falling (95% CI)
Regimen	N/events	1-year OS (%)	Median OS (months)	N/events	1-year OS (%)	Median OS (months)	
**All**	1124/673	56.1 (53.1–59.2)	15.7 (13.8–17.6)	1177/625	68.9 (66.1–71.7)	21.5 (19.9–25.2)	1.36 (1.22–1.53)
**NSCLC**							
Pembrolizumab/atezolizumab with ChT (first line)	136/76	61.4 (53.3–70.7)	17.7 (13.1–21.3)	227/123	66.5 (60.3–73.2)	17.5 (15.1–21.3)	1.14 (0.86–1.53)
Atezolizumab/pembrolizumab (first line)	122/79	50.2 (41.7–60.5)	12.2 (8.9–20.3)	186/102	68.7 (62.1–75.9)	28.1 (20.4–38.9)	1.67 (1.24–2.25)
Atezolizumab/pembrolizumab (second line)	102/88	43.5 (34.7–54.5)	9.1 (8.0–13.5)	77/63	56.1 (46.0–68.6)	14.2 (10.1–19.7)	1.52 (1.10–2.11)
**Melanoma**							
Nivolumab and Ipilimumab (first or second line)	157/59	68.8 (61.6–76.7)	NA (34.0-NA)	139/49	77.9 (71.0–85.5)	NA (30.3-NA)	1.19 (0.81–1.74)
Nivolumab/pembrolizumab (first or second line)	133/63	69.5 (61.8–78.1)	28.6 (18.8–50.0)	103/52	82.7 (75.6–90.6)	35.6 (23.8–52.6)	1.17 (0.81–1.71)
**Head and neck**							
Nivolumab/pembrolizumab (first or second line)	130/101	32.8 (25.2–42.8)	8.1 (6.4–10.5)	92/54	59.4 (49.8–70.8)	13.9 (11.7–18.4)	1.84 (1.32–2.58)
**SCLC**							
Atezolizumab with ChT (first line)	51/36	28.7 (17.9–46.0)	9.0 (7.9–10.8)	102/70	55.4 (45.9–67.0)	13.1 (10.7–14.5)	1.36 (0.89–2.08)
**Renal**							
Avelumab/pembrolizumab with TKI (first line)	57/17	78.7 (68.3–90.8)	NA (35.2-NA)	101/30	82.8 (75.2–91.2)	41.0 (28.8-NA)	0.79 (0.43–1.44)
Nivolumab (second line)	109/74	62.2 (53.6–72.2)	16.4 (12.6–22.1)	39/29	54.7 (40.8–73.3)	14.6 (8.8–28.9)	0.81 (0.52–1.26)
Nivolumab and ipilimumab (first line)	71/35	76.6 (67.2–87.4)	34.0 (29.1-NA)	64/29	77.1 (67.2–88.4)	38.6 (26.1-NA)	1.08 (0.66–1.78)
**Urothelial**							
Atezolizumab/pembrolizumab (second line)	56/45	27.7 (17.8–43.2)	6.1 (4.2–8.2)	47/24	64.9 (51.5–81.8)	26.8 (12.8–38.6)	2.88 (1.71–4.86)

aHR, adjusted hazard ratio; ChT, chemotherapy; CI, confidence interval; NSCLC, non-small cell lung cancer; OS, overall survival; SCLC, small cell lung cancer; SIII, systemic immune-inflammation index; TKI, tyrosine kinase inhibitor.

**Figure 3.**
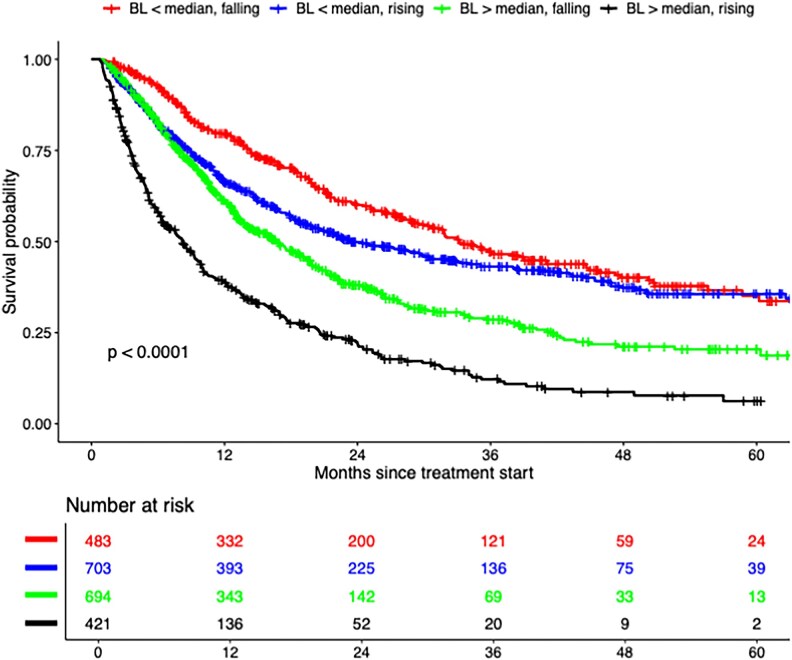
OS according to baseline (BL) and on-treatment systemic immune-inflammation index (SIII) among patients receiving immune checkpoint inhibitors.

There was a significant interaction between baseline SIII and on-treatment SIII with respect to OS (*P* < 0.001). Patients with below-median baseline SIII and an on-treatment fall (reference group) had the most favourable outcomes (median OS 33.2 months, 29.5–41.0; 1-year OS 79.6%, 75.9–83.4) (Table 4, Fig. 3). Patients with below-median baseline SIII but an on-treatment rise had a median OS of 23.4 months (19.9–30.5) and a 1-year OS of 66.2% (62.7–70.0) (aHR 1.31, 1.11–1.56), which was comparable to patients with above-median baseline SIII and an on-treatment fall (median OS 16.7 months, 14.2–19.1; 1-year OS 61.2%, 57.5–65.1) (aHR 1.61, 1.36–1.91). The survival curves for these two groups were similar during the first 12 months but then diverged, with the group with below-median baseline SIII showing substantially better long-term outcomes, though the value of direct comparison is limited by heterogeneity. Patients with above-median baseline SIII and an on-treatment rise had the poorest outcomes, with a median OS of 8.2 months (6.6–9.6) and a 1-year OS of 38.9% (34.3–44.1) (aHR 2.88, 2.41–3.44).

**Table 4.** Overall survival by SIII at baseline and at 3–6 weeks after treatment initiation.

	N/events	1-year OS (%)	Median OS (months)	aHR (95% CI)
Below median baseline SIII, falling on treatment	483/222	79.6 (75.9–83.4)	33.2 (29.5–41.0)	1
Below median baseline SIII, rising on treatment	703/353	66.2 (62.7–70.0)	23.4 (19.9–30.5)	1.31 (1.11–1.56)
Above median baseline SIII, falling on treatment	694/403	61.2 (57.5–65.1)	16.7 (14.2–19.1)	1.61 (1.36–1.91)
Above median baseline SIII, rising on treatment	421/320	38.9 (34.3–44.1)	8.2 (6.6–9.6)	2.88 (2.41–3.44)

aHR, adjusted hazard ratio; ChT, chemotherapy; CI, confidence interval; OS, overall survival; SIII, systemic immune-inflammation index.

## Discussion

This large retrospective cohort study included 2578 patients with a median follow-up of 2.6 years, during which there were 1514 OS events and examined the effect of SIII on OS in patients receiving ICIs at a major UK cancer centre. A baseline SIII below the median was associated with significantly longer OS for the entire cohort (aHR 0.56, 0.50–0.62) and across almost all cancer types and treatment regimens. An on-treatment rise in SIII was associated with worse OS (aHR 1.36, 1.22–1.53), and there was a significant interaction between baseline SIII and on-treatment changes. Patients with low baseline SIII that further decreased on treatment experienced the longest OS, whereas those with a high baseline SIII that increased on treatment had the poorest OS (aHR 2.88, 2.41–3.44).

Our findings suggest that SIII may serve as a low-cost biomarker for patients receiving ICIs, with both baseline levels and on-treatment changes providing valuable insights. A low baseline SIII may reflect a more favourable immune microenvironment, potentially associated with higher lymphocyte activity and reduced inflammatory signalling, which could support a better response to immune checkpoint inhibitors [14]. In contrast, a high SIII may indicate a more inflamed and immunosuppressive state, potentially driven by neutrophils and platelet-derived factors such as TGF-β, which have been shown to impair cytotoxic immune function and promote immune evasion [15, 16]. Used alongside other prognostic factors, SIII could support more individualized estimates of prognosis in clinical practice, though specific thresholds still need to be defined.

Beyond prognostication, these results suggest that SIII has potential to help guide treatment selection in certain cancer types. In renal cancer, patients with above-median SIII achieved comparable OS to those with below-median SIII when treated with TKI–ICI combinations, but experienced substantially worse OS with nivolumab plus ipilimumab. A smaller but consistent effect was observed in NSCLC, where high SIII was more detrimental with ICI monotherapy than with ICI–chemotherapy. This pattern may be due to the direct anti-tumour effects of TKIs and chemotherapy, which rely less on immune modulation [17, 18]. Prospective studies that adjust for established prognostic factors [e.g. programmed death-ligand 1 (PD-L1) in NSCLC and International Metastatic RCC Database Consortium risk in renal cancer] are needed to confirm the role of SIII in regimen selection where multiple options exist.

In this study, on-treatment increases in SIII were associated with worse survival, though the effect on OS was less pronounced than that of baseline SIII and observed only in some cancer types and treatment regimens. A significant interaction was found between baseline SIII and on-treatment changes, with smaller effects when baseline SIII was already low. Conversely, a rise in SIII was most detrimental in patients with high baseline SIII, who already had poorer prognosis even after adjusting for cancer type and therapy. Depending on the cancer type and treatment regimen, these findings suggest that patients with rising on-treatment SIII may require closer monitoring for early treatment failure.

A number of previous studies have investigated the effect of SIII in patients receiving ICIs. In Canada, Miller et al. [19] studied 30 patients with metastatic melanoma receiving ipilimumab and carboplatin/paclitaxel, finding that baseline SIII ≥1375 was associated with worse OS (HR 3.30, 1.21–8.96). In Italy, De Giorgi et al. [20] conducted a study with 313 patients with renal cancer treated with nivolumab and similarly found that baseline SIII ≥1375 was associated with poorer OS (HR 2.99, 2.07–4.31). In China, Liu et al. [21] investigated 44 patients with metastatic NSCLC receiving nivolumab, finding that elevated SIII at baseline predicted worse progression-free survival (PFS) (HR 2.45, 1.67–3.59). Another study in China by Chen et al. [22] examined 139 patients with advanced gastric cancer treated with PD-1/PD-L1 inhibitors, also finding that high baseline SIII was associated with worse OS and PFS (HR 1.99, 1.23–3.23). Additionally, that study found that SIII at 6 weeks was strongly associated with OS (HR 2.78, 1.64–4.70). In a multi-country study, Rebuzzi et al. [23] investigated 571 patients with metastatic renal cancer receiving nivolumab, reporting that higher baseline SIII levels were significantly associated with worse OS and PFS. Other studies have proposed models that combine routinely measured baseline tests similar to SIII with genomic data (e.g. LORIS) to accurately predict ICI outcomes across multiple cancer types [24].

The present study has several strengths. First, it is the largest cohort study to evaluate the role of SIII, including more than 2500 patients with a large number of survival events across multiple cancer types and therapies. The associations between SIII and real-world survival outcomes in commonly used treatment regimens were estimated, providing directly applicable insights for clinical practice. The interaction between baseline and on-treatment SIII was also evaluated, identifying distinct prognostic subgroups. Furthermore, key factors such as age, sex, and performance status (PS), were adjusted for in multivariable regression analyses, reducing potential confounding.

However, there are also some important limitations. Despite the large sample size, substantial heterogeneity in cancer types and treatment regimens limited the comparability of descriptive estimates (e.g. survival times or percentages) according to baseline or on-treatment SIII across the overall population. The lack of a predefined threshold for SIII meant that the median pre-treatment SIII was used to categorize patients, resulting in groups of equal size but without a biologically or clinically validated cut-off. The availability of later lines of treatment after progression on ICIs, which likely varied substantially between patients, was also not accounted for. Some subgroup analyses were limited by small sample sizes, reducing statistical power to detect associations. Our analyses of on-treatment changes in SIII may have been affected by bias from immortal time between treatment initiation and SIII measurement. The timing of on-treatment SIII measurements varied between patients and may have been influenced by factors related to disease progression. Although we adjusted for known confounders, unmeasured confounding variables may still have influenced the results. We did not adjust a significance threshold that accounted for multiple testing and, as a result, some of the observed associations may have been due to chance. Finally, while our findings demonstrate a strong association between SIII and OS, prospective clinical evidence is needed to support its use in decision-making.

A further limitation is the potential use of granulocyte colony-stimulating factor (G-CSF) or corticosteroids, which was unknown and may have influenced neutrophil counts and thus SIII. This would primarily have affected the evaluation of on-treatment SIII. Although G-CSF was not routinely administered, a small number of patients receiving chemo-immunotherapy for NSCLC or SCLC may have received it for ≥grade 3 neutropenia (e.g. in the context of infection). Corticosteroids were routinely used as supportive medication with chemo-immunotherapy (3 days of dexamethasone) and may also have been given for symptom control in progressive disease or for management of immune-related adverse events, which have been associated with improved outcomes in some cancers [25, 26]. The net effect of these medications may have attenuated the SIII–OS association if they were used for reasons unrelated to prognosis; conversely, if their use was linked to prognosis, the association could be biased in either direction.

In summary, this study provides evidence that SIII has the potential to be a valuable biomarker for patients receiving ICIs. Baseline SIII was strongly associated with OS, while early on-treatment changes in SIII provided additional insight for some cancer types and treatment regimens, potentially enabling earlier identification of patients who may not be responding to treatment. These findings suggest potential for SIII to be incorporated into clinical practice to help stratify patients, guide treatment decisions, and identify those who may benefit from closer monitoring. However, further prospective studies and clinical trials are required before specific recommendations can be made.

## Supplementary Material

ltaf031_Supplementary_Data

## Data Availability

No additional data are available.
