# Supplementary material for: Overall survival by baseline and on-treatment systemic immune-inflammation index in patients with advanced cancer receiving immune checkpoint inhibitors: a large single-centre cohort study
Source: Immunother Adv. 2026 Feb 27;6(1):ltaf031. doi: 10.1093/immadv/ltaf031 (PMC13023032; doi:10.1093/immadv/ltaf031)
Supplement: ltaf031_Supplementary_Data [file ltaf031_supplementary_data.docx]

**Overall Survival by Baseline and On-Treatment Systemic Immune-Inflammation Index in Patients with Advanced Cancer Receiving Immune Checkpoint Inhibitors: A Large Single-Centre Cohort Study – supplementary information**

**Oliver John Kennedy^1,2*^, Rebecca Lee^1,2^, Fiona Blackhall^1,2^,**  [**Ananya Choudhury**](mailto:ananya.choudhury@nhs.net)**^1,2^, Robert Metcalf^2,3^, Tom Waddell^2^, Paul Lorigan^1,2^**

^1^Division of Cancer Sciences, University of Manchester, Manchester, United Kingdom

^2^Christie NHS Foundation Trust, Manchester, Wilmslow Rd, Manchester M20 4BX, United Kingdom

^3^Division of Immunology, Immunity to Infection and Respiratory Medicine, University of Manchester, Manchester, United Kingdom

**Supplementary Table 1**. Median Systemic Immune-Inflammation Index

| **Treatment group** | **Median SIII** |
| --- | --- |
| **All** | 1316 |
| **NSCLC** |  |
| Pembrolizumab/atezolizumab with ChT (1st line) | 2028 |
| Atezolizumab/pembrolizumab (1st line) | 1851 |
| Atezolizumab/pembrolizumab (2nd line) | 1341 |
| **Melanoma** |  |
| Nivolumab and Ipilimumab (1st or 2nd line) | 923 |
| Nivolumab/pembrolizumab (1st or 2nd line) | 886 |
| **Head and neck** |  |
| Nivolumab /Pembrolizumab (1st or 2nd line) | 2025 |
| **SCLC** |  |
| Atezolizumab with ChT (1st line) | 1331 |
| **Renal** |  |
| Avelumab/pembrolizumab with TKI (1st line) | 950 |
| Nivolumab (2nd line) | 1066 |
| Nivolumab and ipilimumab (1st line) | 1107 |
| **Urothelial** |  |
| Atezolizumab/pembrolizumab (2nd line) | 1291 |

**Abbreviations**: Systemic Immune-Inflammation Index (SIII), NSCLC (non-small cell lung cancer), SCLC (small cell lung cancer), TKI (tyrosine kinase inhibitor), SIII (systemic immune-inflammation index), OS (overall survival), ChT (chemotherapy)

**Supplementary Figures**


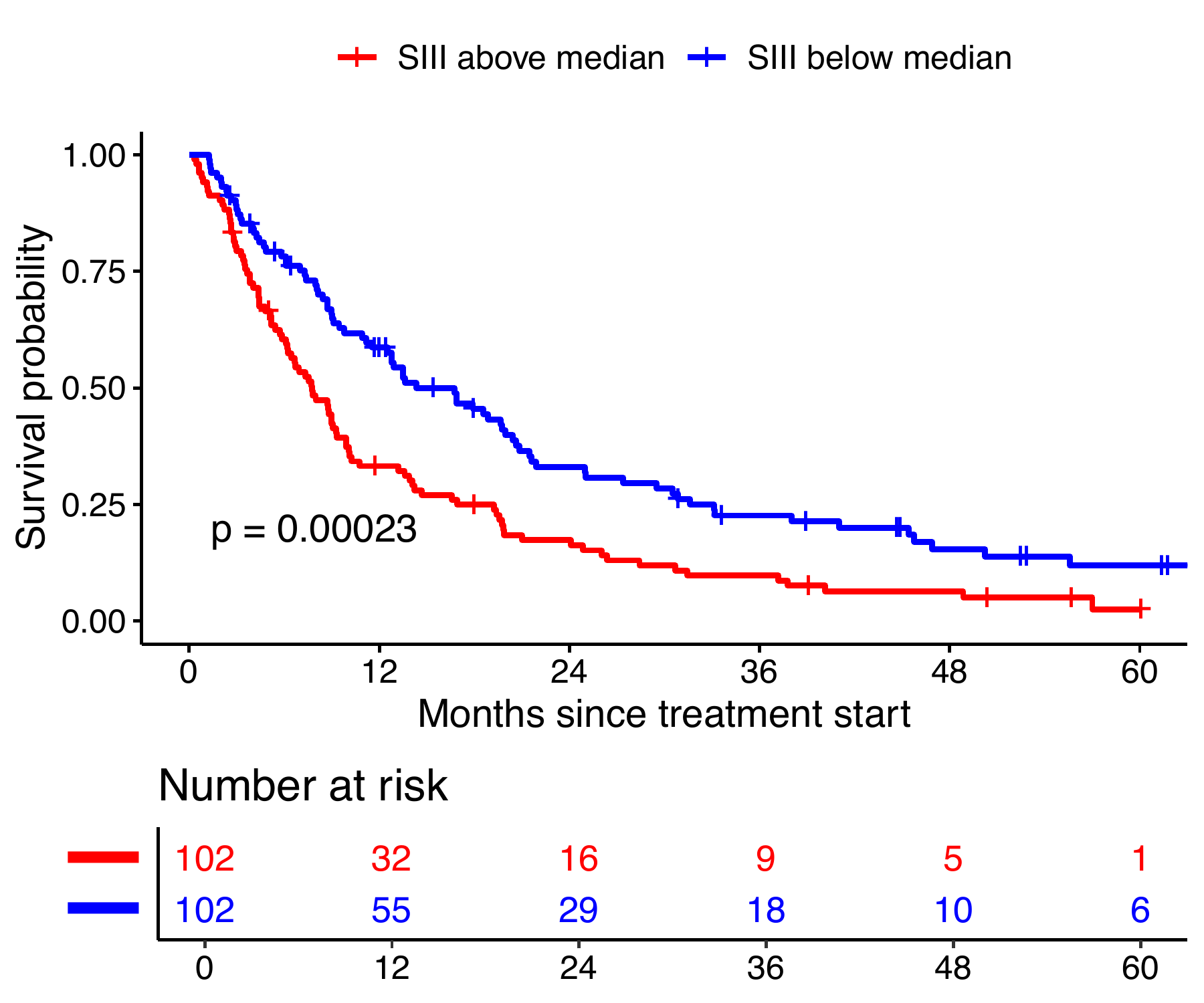


**Supplementary Figure 1**. Overall survival among patients with non-small cell lung cancer receiving atezolizumab or pembrolizumab monotherapy (2nd line), stratified by baseline SIII above vs. below the median.


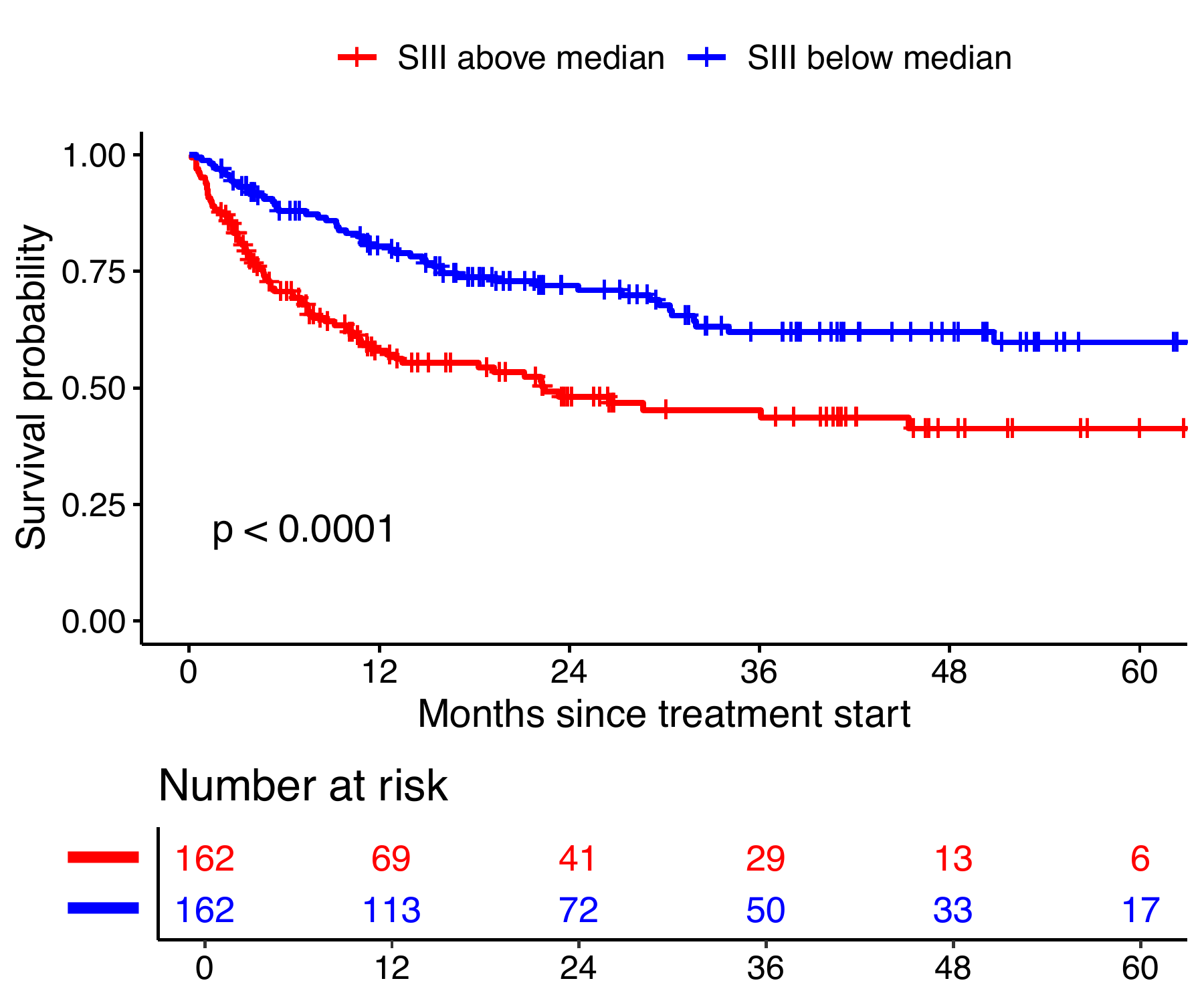


**Supplementary Figure 2**. Overall survival among patients with melanoma receiving nivolumab and ipilimumab, stratified by baseline SIII above vs. below the median.


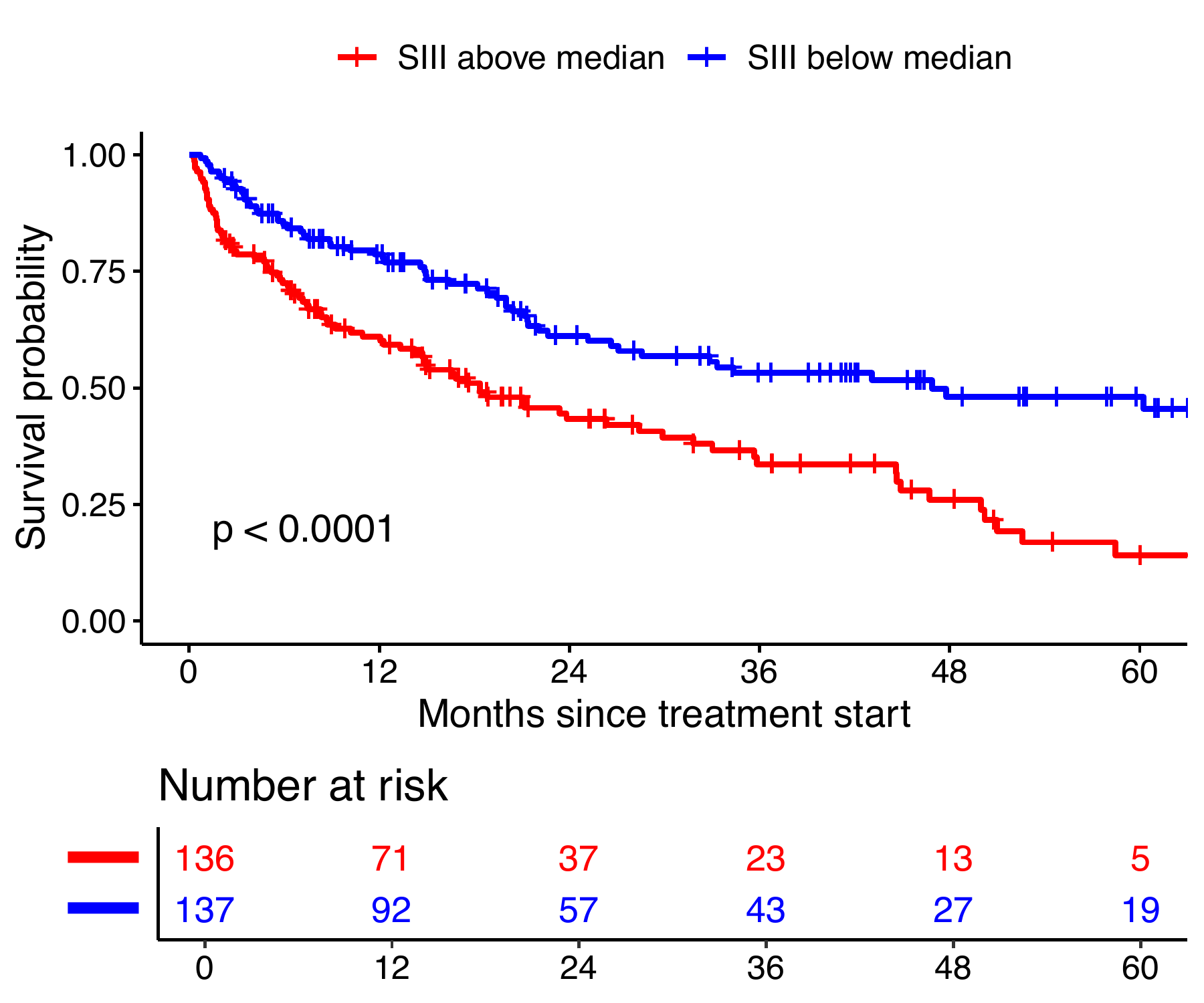


**Supplementary Figure 3**. Overall survival among patients with melanoma receiving nivolumab or pembrolizumab monotherapy, stratified by baseline SIII above vs. below the median.


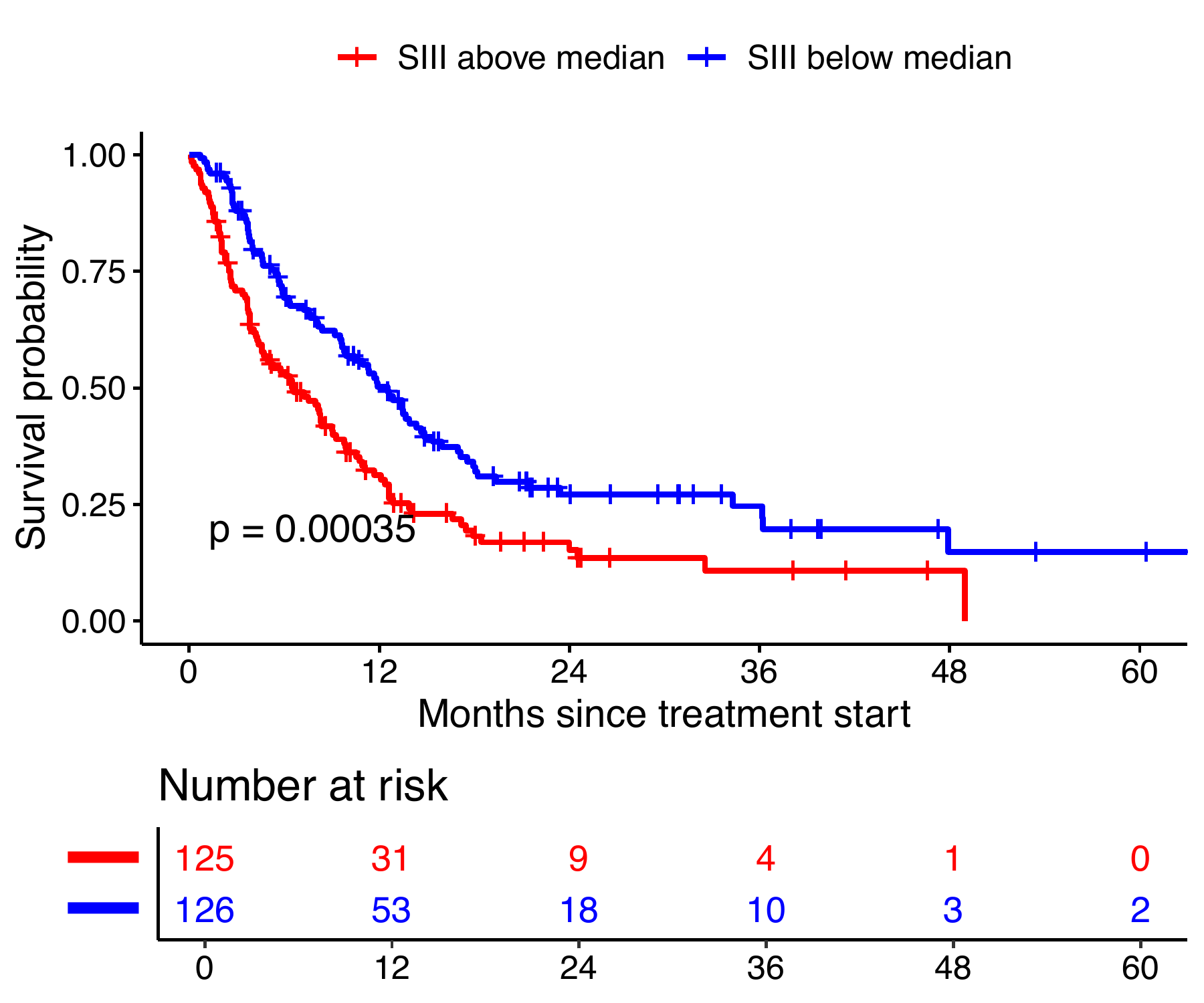


**Supplementary Figure 4**. Overall survival among patients with head and neck cancer receiving nivolumab or pembrolizumab (1st or 2nd line), stratified by baseline SIII above vs. below the median.


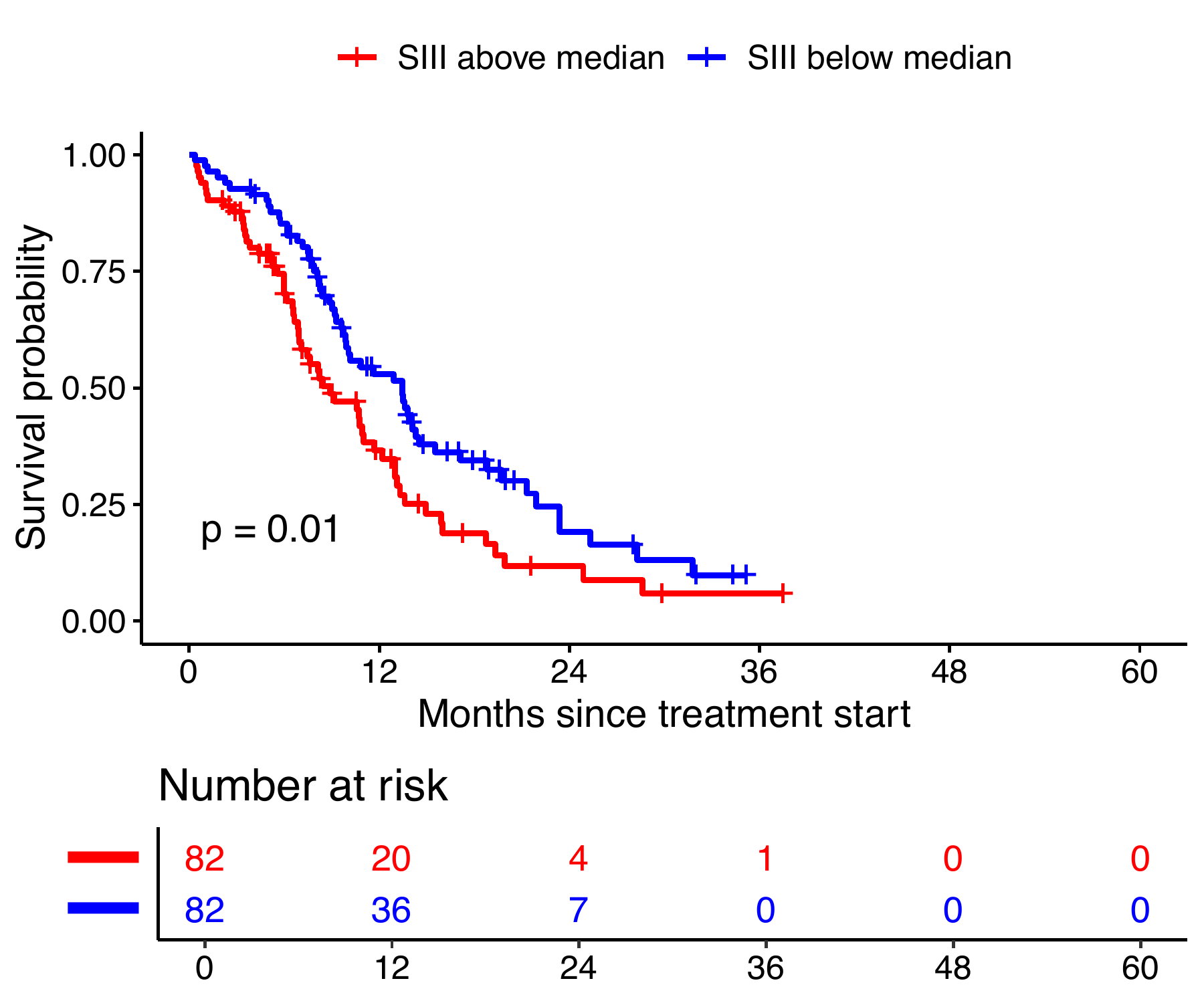


**Supplementary Figure 5**. Overall survival among patients with small cell lung cancer receiving atezolizumab with chemotherapy (1st line), stratified by baseline SIII above vs. below the median.


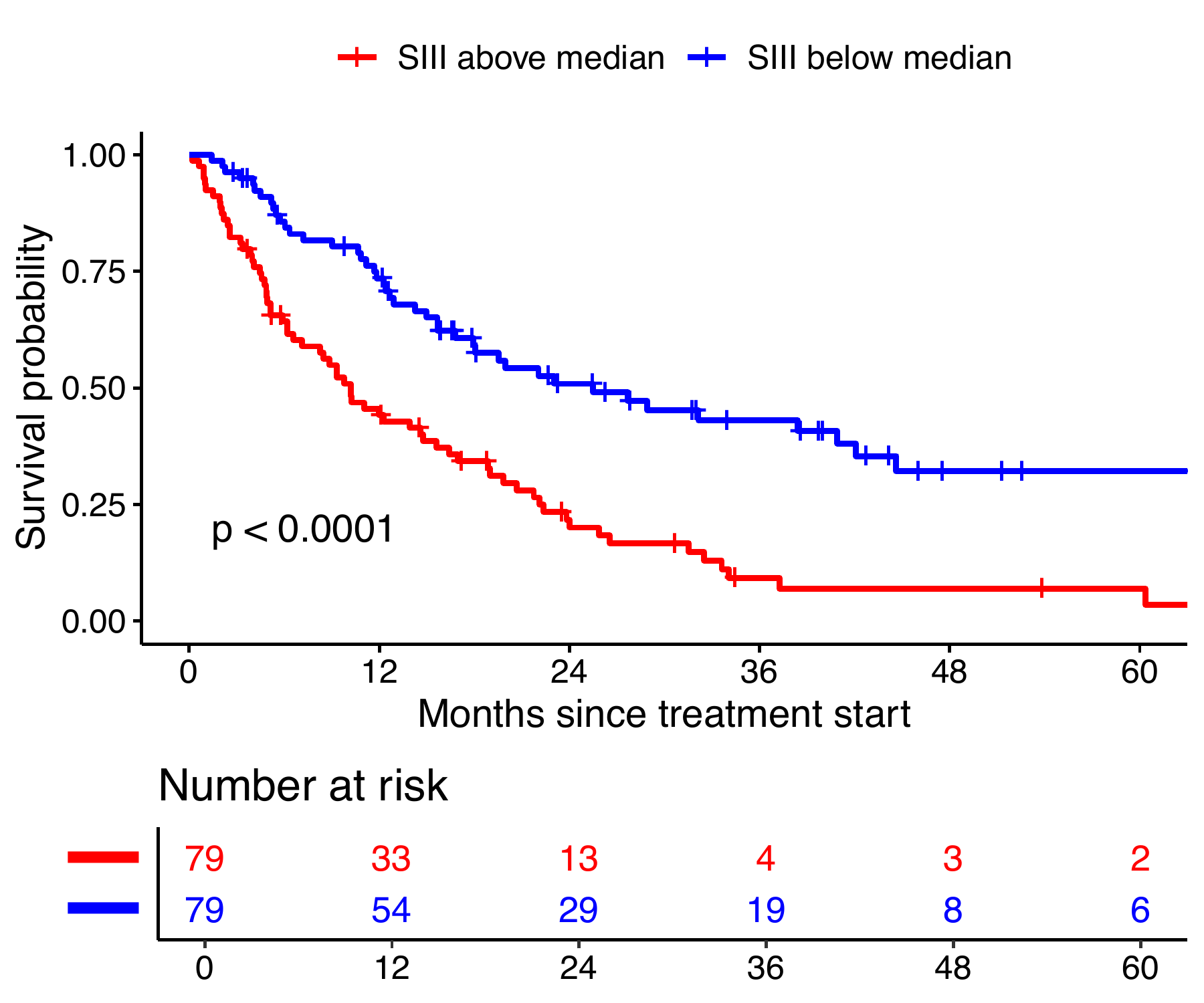


**Supplementary Figure 6**. Overall survival among patients with renal cell carcinoma receiving nivolumab monotherapy (2nd line), stratified by baseline SIII above vs. below the median.


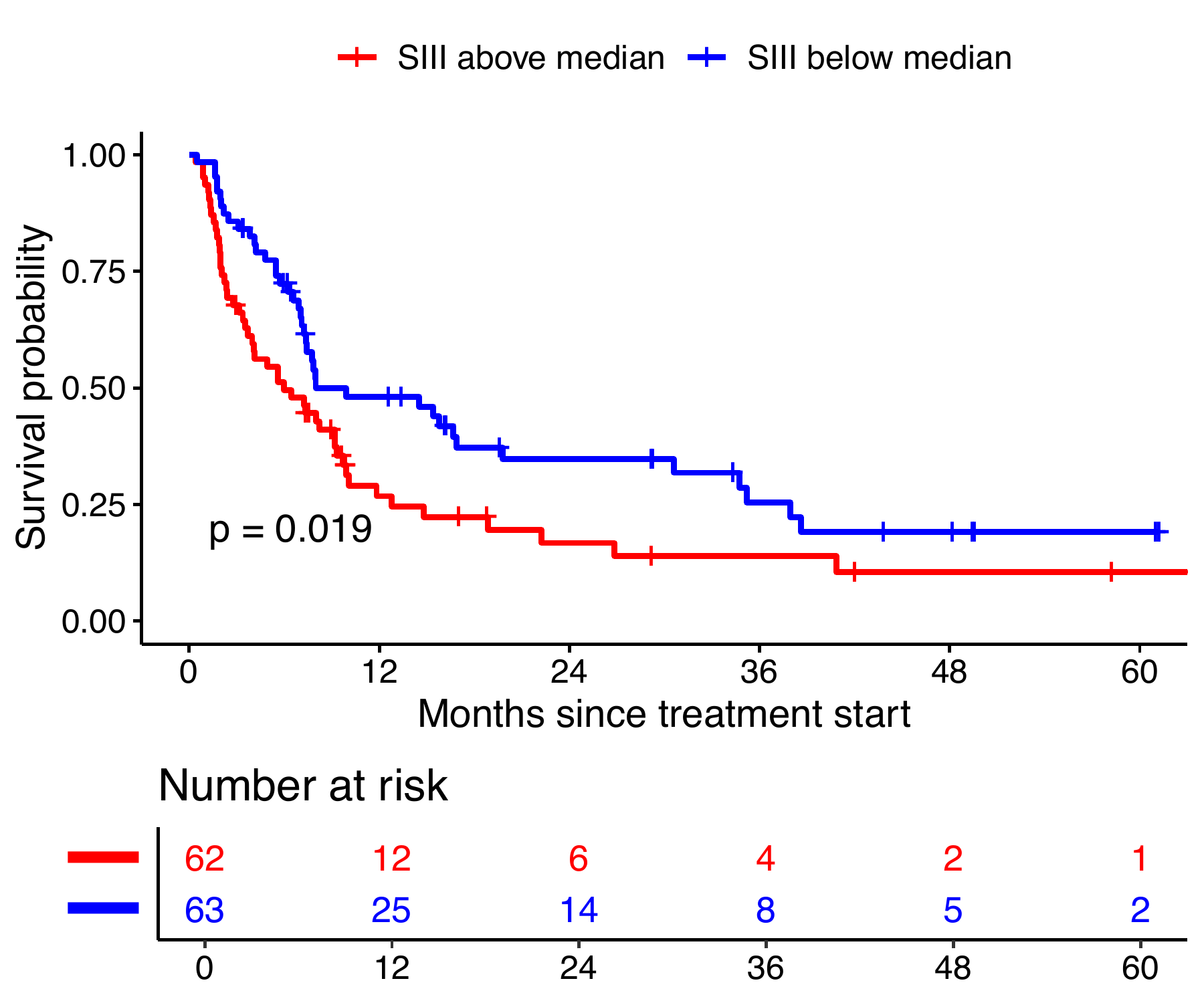


**Supplementary Figure 7**. Overall survival among patients with urothelial carcinoma receiving atezolizumab or pembrolizumab (2nd line), stratified by baseline SIII above vs. below the median.
